# Effect of Short-Term and Long-Term Non-Physiological T3 Concentrations on Cardiac Stromal Cells: From Cellular Response to In Vivo Adaptation

**DOI:** 10.3390/medsci14010066

**Published:** 2026-01-31

**Authors:** Ahmad Alhamid, Yoshishige Urata, Kodai Nishi, Hiroshi Kurazumi, Ryo Suzuki, Koji Ueno, Akihito Mikamo, Kimikazu Hamano, Tao-Sheng Li

**Affiliations:** 1 Department of Stem Cell Biology, Nagasaki University Graduate School of Biomedical Sciences, 1-12-4 Sakamoto, Nagasaki 852-8523, Japan; 2 Department of Stem Cell Biology, Atomic Bomb Disease Institute, Nagasaki University, 1-12-4 Sakamoto, Nagasaki 852-8523, Japan; jj20250126@ms.nagasaki-u.ac.jp; 3 Department of Radioisotope Medicine, Atomic Bomb Disease Institute, Nagasaki University, 1-12-4 Sakamoto, Nagasaki 852-8523, Japan; koudai@nagasaki-u.ac.jp; 4 Department of Surgery and Clinical Science, Yamaguchi University Graduate School of Medicine, 1-1-1 Minami-kogushi, Yamaguchi, Ube 755-8505, Japan; kurazumi@yamaguchi-u.ac.jp (H.K.); ryo-s@yamaguchi-u.ac.jp (R.S.); kjueno@yamaguchi-u.ac.jp (K.U.); mikamo@yamaguchi-u.ac.jp (A.M.); kimikazu@yamaguchi-u.ac.jp (K.H.)

**Keywords:** cardiac fibroblast, cardiac stromal cell, thyroid hormone, hyperthyroid, hypothyroid

## Abstract

**Background/Objectives:** Epidemiological and clinical studies have linked both hypothyroidism and hyperthyroidism to adverse cardiac outcomes, including heart failure and myocardial fibrosis. Triiodothyronine (T3), a biologically active thyroid hormone, is important for cardiovascular homeostasis. While the effects of physiological and non-physiological T3 levels on cardiomyocytes have been extensively investigated, the impact of hypothyroidism and hyperthyroidism on cardiac stromal cells (CSCs), which constitute the majority of the cells in the heart, remains understudied. Given CSCs’ essential role in extracellular matrix (ECM) remodeling and paracrine signaling, understanding their response to altered T3 states is necessary to fully elucidate the thyroid hormone-induced cardiac responses. **Methods**: Cardiac stromal cells were isolated from human atrial appendages and cultured under hypothyroid (0 nM T3), euthyroid (2.5 nM T3), and hyperthyroid (25 nM T3) conditions for 24 (short term) and 120 h (long term). The cells were harvested after 24 h of treatment using trypsin and automatically counted, and their ECM-related gene and growth factor expression levels were assessed using quantitative RT-PCR. Cardiac glucose uptake in hypothyroid, euthyroid, and hyperthyroid mice was monitored using [18F]-FDG PET/CT at acute (7 days) and chronic (42 days) time points. **Results**: Both hypo- and hyperthyroidism significantly increased the number of CSCs harvested after 24 h. There were acute alterations in the expression of the ECM-related genes COL1A1, COL3A1, TIMP3 (*p* < 0.05), and TIMP1 (*p* < 0.01). Similarly, growth factors such as PDGF-A (*p* < 0.001), TGF-b, and IGF1 (*p* < 0.05) were transiently upregulated under non-physiological T3 conditions, especially hypothyroidism. Most of these alterations were attenuated or reversed at the 120 h time point. In vivo PET imaging revealed significant increases in cardiac glucose uptake under acute hypothyroidism (*p* < 0.05) and decreases under acute hyperthyroidism (*p* < 0.05). However, these metabolic shifts normalized with chronic exposure, paralleling the transient nature of the gene expression changes observed in vitro. **Conclusions**: Non-physiological T3 concentrations induce proliferation and changes in ECM-related and growth factor gene expression in CSCs. Most of these changes are acute and return to normal levels after chronic exposure. These transient cellular responses correlate closely with the cardiac metabolic response patterns to acute and chronic hypothyroidism and hyperthyroidism.

## 1. Introduction

The physiologically active thyroid hormone triiodothyronine (T3) affects cardiac development, function, and adaptation in both healthy and diseased conditions, making it essential for cardiovascular homeostasis. Strong epidemiological evidence links subclinical low-T3 states and hypothyroidism to higher cardiovascular morbidity and mortality, such as a higher risk of heart failure, coronary artery disease, and worsened myocardial recovery after ischemic injury [1]. Clinical studies show a worse prognosis in heart failure patients with lower serum T3 levels [2]. A meta-analysis showed that thyroid hormone replacement therapy can effectively treat patients with heart failure and low-triiodothyronine syndrome by improving the left ventricular ejection fraction, cardiac output, and early-to-late diastolic trans mitral flow velocity. It also decreased the brain natriuretic peptide and noradrenaline levels [3]. On the other hand, hyperthyroidism results in elevated cardiac output and left ventricular hypertrophy in the initial phase, progressing to biventricular dilation and congestive heart failure in the latter phase. Atrial fibrillation and pulmonary arterial hypertension contribute to the high morbidity associated with untreated hyperthyroidism [4]. Heart failure resulting from hyperthyroidism has been regarded as a reversible etiology of cardiomyopathy. Numerous reports indicate that patients with symptomatic heart failure exhibit a reversal of echocardiographic measurements and clinical symptoms upon achieving a euthyroid state [5,6]. Both hyperthyroidism and hypothyroidism induce cardiac fibrosis [7,8], while physiological levels of T3 inhibit pathological fibrosis [9].

The effects of thyroid hormones on cardiomyocytes have been thoroughly described. Physiological concentrations of T3 exert pro-survival, anti-apoptotic effects on cardiomyocytes [10,11] (Nicolini et al., 2013) (Ojamaa 2010). Additionally, physiological T3 levels support respiratory efficiency and mitochondrial biogenesis in cardiomyocytes; this support is compromised in thyroid hormone deficiency [10] (Nicolini et al., 2013). Hyperthyroidism increases mitochondrial free radical production and diminishes the cellular antioxidant capacity in cardiomyocytes, leading to oxidative stress [12] (Araujo et al., 2011). Interestingly, Weltman et al., [13] (Weltman et al., 2014) found that chronic hyperthyroidism is associated with normal or enhanced mechanical cardiomyocyte function despite declines in cardiac mechanical parameters, pointing to the possibility that T3’s impact on the heart might be due to its effects on non-cardiomyocyte cells.

Insights from stromal cells in organs other than the heart highlight the necessity of examining T3’s effects on cardiac stromal cells. Physiological T3 concentrations prevent the production of collagen [14], fibronectin [15], and glycosaminoglycans [16] by dermal fibroblasts. Thyroid hormones also activate angiogenic signaling through integrin αvβ3 in cancer mesenchymal stem cells [17] (Schmohl et al., 2019). Crucially, Safer et al., [18] (Safer et al., 2003) showed that although T3 promotes keratinocyte proliferation, fibroblasts can counteract this effect when co-cultured with keratinocytes. This points to the importance of incorporating stromal cells when studying the effect of T3 on organs.

Cardiac fibroblasts make up between 60 and 70 percent of the heart’s cells. They play a key role in controlling cardiac remodeling through their functions in paracrine signaling, extracellular matrix turnover, and mechanical property modulation [19] (Tallquist and Molkentin 2017). However, most of the current evidence regarding T3’s role in cardiac physiology is myocyte-centric, with stromal cell contributions being noticeably understudied [20] (Yamakawa et al., 2021).

We propose that hypothyroidism and hyperthyroidism modulate cardiac physiology by affecting extracellular matrix production and growth factor secretion from cardiac stromal cells (CSCs). The proteins produced by CSCs feed back onto cardiomyocytes, inducing cardiac responses and adaptation to the altered thyroid status.

Thus, we evaluated the effects of T3 on proliferation and extracellular matrix-related and growth factor gene expression in cardiac stromal cells under short- and long-term hypothyroid, euthyroid, and hyperthyroid conditions. We also used serial cardiac positron-emission tomography (PET) on mice subjected to acute and chronic hypothyroidism, euthyroidism, and hyperthyroidism to visualize the metabolic response to connect our findings to organ-level physiological changes.

## 2. Materials and Methods

**Ex Vivo Expansion of Cardiac Stromal Cells**

Left atrial appendages were excised from patients receiving heart surgery at Yamaguchi University Hospital after they provided informed consent. Ethical approval was given by the Yamaguchi University Graduate School of Medicine (code: H2021-126; date: 4 October 2021) and Nagasaki University Atomic Bomb Disease Institute (code: G22080501; date: 26 July 2022). The atrial appendages were sent in DMEM supplemented with 10% fetal bovine serum (FBS) at 4 °C. The expansion of cardiac stromal cells was accomplished using the protocol described in [21] (Li et al., 2010). Briefly, the atrial tissues were chopped into tiny pieces and explanted on plastic dishes coated with 15 μg/mL fibronectin (CORNING, Corning, NY, USA). Stromal-like cells grew out of the explants and became confluent 10–14 days later. These cells were further passaged on plastic dishes, and the cells from different patients were pooled. All cells were cultured in basic IMDM (Gibco, Thermo Fisher Scientific, Waltham, MA, USA) supplemented with 10% FBS.

**In Vitro T3 Treatment and Cell Quantification**

At passage 2–3, the medium was exchanged for basic IMDM supplemented with 10% charcoal-stripped FBS. Approximately 250 k cells were seeded in 6 cm dishes for the 24 h group, and 150 k cells were seeded for the 120 h group. For the 24 h group, the treatment started once the cells reached 70–80% confluence, while for the 120 h group, treatment was started early considering the long treatment time; the cells usually reached approximately 80% confluence by the end of the experiment. The medium was supplemented with 0, 2.5, or 25 nM T3 (Sigma-Aldrich, Tokyo, Japan T6397), which represent hypothyroidism, euthyroidism, and hyperthyroidism conditions. The T3 concentrations were selected based on physiological reference ranges. The reference range for total T3 concentration in humans is 1.1–2.9 nM, as documented in the *Williams Textbook of Endocrinology* [22] (Larsen PR 2003). We selected the midpoint of this physiological range (2.5 nM) as the euthyroid concentration. The hypothyroid concentration (0 nM T3) represents complete T3 deprivation. The hyperthyroid concentration (25 nM) represents a 10-fold supraphysiological dose. Schmohl et al., [17] used the same approach for cancer-associated mesenchymal cells (Schmohl et al., 2019). Lars C Moeller used 0 nM vs. 2 nM total T3 concentration in an in vitro study on T3-responsive genes in fibroblasts [23] (Moeller et al., 2005). T3 was dissolved in 1 N NaOH to prepare a 1 mM stock solution, which was further diluted in culture medium to achieve the final working concentrations. The stock solution was stored at −20 °C and used within one month of preparation. The final NaOH concentration was ≤0.025 mM.

The cells were treated for 24 h for the short-term treatment and 120 h for the long-term treatment. In the long-term treatment, the media was changed every other day. After the treatment was finished, the cells were lysed in situ using the lysis buffer of an RNA extraction kit or harvested by incubating with 0.25% trypsin for five minutes. The cells were then counted using a BioRad TC20 automatic cell counter.

**Reverse Transcription–Quantitative Polymerase Chain Reaction**

RNA was isolated from cells using the Direct-zol™ RNA MiniPrep Kit (Zymo Research, Tustin, CA, USA) following the manufacturer’s protocol. The quantity and quality of the RNA were evaluated using a NanoDrop™ 2000/2000c spectrophotometer (Thermo Scientific, Waltham, MA, USA). RNA samples with an A260/A280 ratio between 1.9 and 2.1 and A260/A230 ratio > 1.8 were considered acceptable for downstream applications. The isolated RNA (1 μg) was reverse transcribed using SuperScript™ VILO™ MasterMix (Invitrogen, Carlsbad, CA, USA) in a 20 μL reaction mixture. Quantitative polymerase chain reaction (qPCR) was performed using THUNDERBIRD™ SYBR^®^ qPCR Mix (TOYOBO, Tokyo, Japan) and a Bio-Rad CFX96 real-time PCR detection system (Bio-Rad, Hercules, CA, USA). Complementary DNA (cDNA) equivalent to 50 ng of RNA was used in each 20 μL PCR reaction. The primers (Table 1) used in this study were synthesized by Hokkaido System Science Co., Ltd. (Tokyo, Japan). Expression was normalized to the expression of the housekeeping gene beta-actin. The normalized fold change was calculated using the ΔΔCq method.

**In vivo hypothyroidism, euthyroidism, and hyperthyroidism models**

Seven-week-old male healthy C57BL/6 mice were used. All experiments were approved by the Institutional Animal Care and Use Committee of Nagasaki University (2107071730-7, date: 18 January 2023). The animals were kept in the Nagasaki University Animal Center at room temperature under 12 h night–dark cycles. Every cage contained a mouse from each group. We anesthetized the mice via an intraperitoneal injection of 4 mg/kg midazolam, 5 mg/kg butorphanol, and 0.75 mg/kg medetomidine. For the hypothyroidism model, total thyroidectomy was performed; for the control mice, a sham operation was performed; and for the hyperthyroidism group, we performed a sham operation and implanted an osmotic mini pump under the back skin to deliver T3 at a dose of 1.5 mcg/kg/day. This dose is equivalent to 100 mcg of T3 per day in human patients with hypothyroidism, which is 2–4 times the usual dose. Adding this dose in addition to the endogenously produced T3 level produced a hyperthyroid state in the mouse. 18F-FDG PET/CT was performed 1 week (short-term) or 6 weeks (long-term) after the operation. We randomly allocated 5 mice to each group; however, one mouse in the long-term hypothyroid group and one mouse in the long-term hyperthyroid group died before imaging. 

**18F-FDG PET/CT imaging**

Imaging was performed using a Triumph LabPETt 4/SPECT4/CT (TriFoil Imaging Inc., Chatsworth, CA, USA), which is a small animal PET/CT scanner. The mice were fasted six hours before imaging. They were injected with 10 MBq of 18F-FDG (Nihon Medi-Physics Co., Kurume, Japan) into their tail vein approximately 30 min before scanning. The mice were scanned for 15 min under anesthesia with 2.0–2.5% isoflurane. For PET imaging data, the 3D maximum-likelihood expectation maximization (3D-MLEM) algorithm was applied using 30 iterations. 18F-FDG uptake was calculated as the mean cardiac uptake per voxel normalized by the mean whole-body uptake per voxel.

**Statistical analysis**

The data is presented as the mean ∓ standard error of the mean (SEM). We used unpaired Student’s *t*-tests to analyze the differences between groups. GraphPad Prism 10 was used to perform all the analyses and produce the figures. We used the Shapiro–Wilk test to assess normality. All datasets showed a *p* value > 0.05, which supports the use of parametric tests. The differences were considered significant if *p* < 0.05.

## 3. Results

**Non-physiological T3 concentrations induce cardiac stromal cell proliferation**

**Figure 1:**
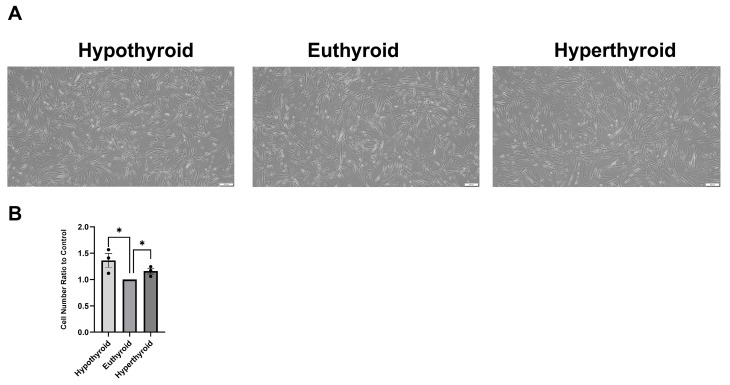
Effect of hypothyroidism and hyperthyroidism on the morphology and number of cardiac stromal cells. (**A**) Representative phase-contrast images of CSCs after 24 h of exposure to low, physiological, and high concentrations of T3. (**B**) Number of harvested cells from each treatment group after 24 h of treatment normalized by number of cells harvested from the control euthyroid group. Data are presented as the mean ± SEM. * *p* < 0.05.

After the CSCs were grown for 24 h under hypothyroidism, euthyroidism, and hyperthyroidism conditions, there were minimal to no significant differences in cell morphology between the three groups (Figure 1A). Compared with the euthyroid group, the number of harvested cells was higher in the hypothyroid (*p* < 0.05) and hyperthyroid (*p* < 0.05) groups, with a more prominent increase in the hypothyroid group (Figure 1B). Collectively, hypo- and hyperthyroidism appear to increase CSC proliferation.

**Effects of short- and long-term hypo- and hyperthyroidism on expression of ECM-related genes in CSCs**

**Figure 2:**
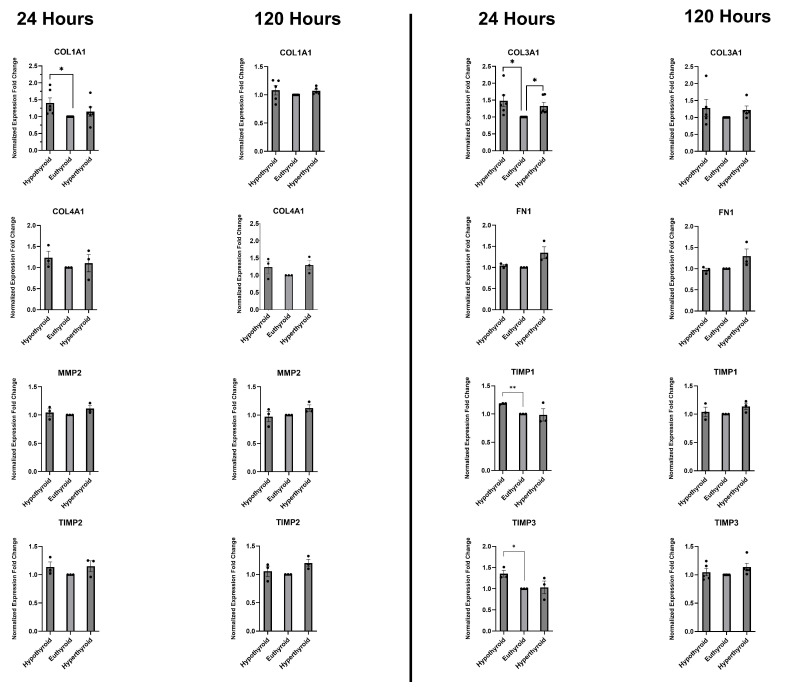
Fold change in gene expression of extracellular matrix components and regulators after 24 h and 120 h of exposure to hypothyroidism, euthyroidism, and hyperthyroidism conditions in vitro. The euthyroid group is the control group. Data are presented as the mean ± SEM. * *p* < 0.05, ** *p* < 0.01.

QRT-PCR analysis showed that non-physiological T3 levels induced changes in the mRNA expression of extracellular matrix (ECM) components and their regulatory genes in CSCs. These changes were dependent on the duration of exposure to the different T3 concentrations (Figure 2). In the short term, there was an increase in *COL1A1* (collagen 1 alpha 1 chain) gene expression in the hypothyroid group (*p* < 0.05) but not in the hyperthyroid group. Over the long term, this increase almost completely disappeared. *COL3A1* (collagen 3 alpha 1 chain) expression increased in the short term in the hypothyroid group (*p* < 0.05); the levels remained high at the 120 h time point but the difference was not statistically significant. *COL4A1* (collagen 4 alpha 1 chain) expression exhibited an upward trend in the low and high T3 groups at 24 and 120 h but the differences were not statistically significant. *FN1* (fibronectin 1) and *MMP2* (matrix metalloproteinase type 2) expression levels were elevated in the supraphysiologic T3 group at both time points but the differences were not statistically significant. The hypothyroid group exhibited marked overexpression of *TIMP1* (tissue inhibitor of metalloproteinases 1) at the 24 h time point (*p* < 0.01); however, at 120 h, this increase disappeared while there was a marginal, non-significant upregulation in the hyperthyroid group. *TIMP2* exhibited a non-significant upregulation in both the hypo- and hyperthyroid groups and at both time points. *TIMP3* expression was markedly elevated in the no T3 group at 24 h (*p* < 0.05); however, this increase did not persist in the long run. Overall, the data demonstrates that low and high T3 levels modulate the gene expression of extracellular matrix (ECM) components and their regulators in cardiac stromal cells, with more prominent effects observed under hypothyroid conditions and at early time points.

**Effects of short- and long-term hypo- and hyperthyroidism on expression of growth factor genes in CSCs**

**Figure 3:**
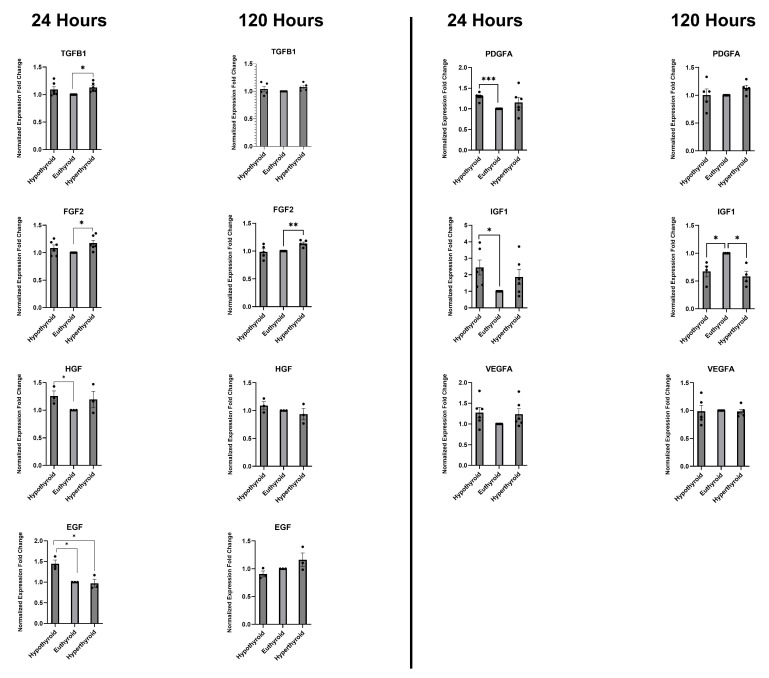
Fold change in gene expression of key growth factors after 24 h and 120 h of exposure to hypothyroidism, euthyroidism, and hyperthyroidism conditions in vitro. The euthyroidism group is the control group. Data are presented as the mean ± SEM. * *p* < 0.05, ** *p* < 0.01, *** *p* < 0.001.

Next, we used qRT-PCR to investigate how non-physiological T3 levels affect the expression of several growth factors genes (Figure 3). TGF-β (transforming growth factor beta) expression was elevated in both the hypo- and hyperthyroid groups compared to the euthyroid group at 24 h, but the difference was only statistically significant for the hyperthyroid group (*p* < 0.05). By 120 h, these differences disappeared. PDGF-A (platelet-derived growth factor subunit alpha) expression was significantly higher in the hypothyroid group compared to the control euthyroid group at 24 h (*p* < 0.001); the increase in the hyperthyroid group was not significant. At 120 h, there were no statistically significant differences in the expression levels of PDGF-A between the groups, although a modest upward trend was observed in the hyperthyroid group. FGF2 (fibroblast growth factor 2) expression levels were higher in both the hypo- and hyperthyroid groups at 24 h, but the difference was only statistically significant for the hyperthyroid group (*p* < 0.05). At 120 h, only the hyperthyroid group maintained significantly elevated FGF2 levels (*p* < 0.01) while the levels in the hypothyroid group were similar to those of the control, indicating a sustained hyperthyroid-associated effect. IGF1 (insulin-like growth factor 1) levels were much higher in both the hypo- and hyperthyroid groups compared to the euthyroid control group after 24 h; the increase in the hypothyroid group was statistically significant (*p* < 0.05). At 120 h, the reverse findings were observed (*p* < 0.05). At 24 h, HGF (hepatocyte growth factor) expression was modestly but significantly increased in the hypothyroid group relative to the euthyroid group (*p* < 0.05), while there was a non-significant increase in the hyperthyroid group. At 120 h, expression differences were no longer apparent except for a minor, insignificant increase in the hypothyroid group. The low-T3 and high-T3 treatment groups saw a significant increase in VEGFA (vascular endothelial growth factor A) expression after 24 h, but this increase was not statistically significant. At 120 h, there were no noticeable differences. The hypothyroid group showed a statistically significant increase in EGF (endothelial growth factor) expression at 24 h (*p* < 0.05); however, only a trend toward increased expression was observed in the hyperthyroid group at 120 h. These findings highlight that deviations from physiological T3 concentrations transiently enhance the expression of multiple growth factor genes in CSCs, but prolonged thyroid hormone imbalance can diminish these increases and selectively downregulate *IGF1* expression.

**Cardiac glucose uptake shows early divergence and long-term normalization in response to non-physiological thyroid conditions**

**Figure 4:**
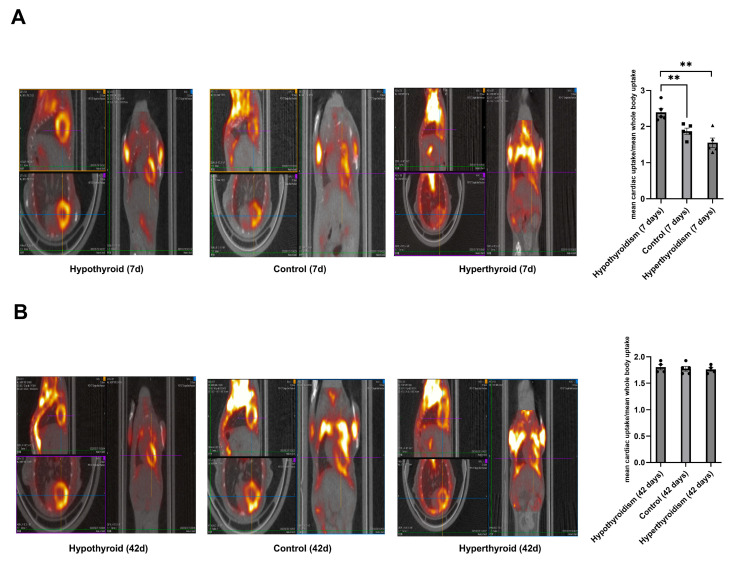
[^18^F]-FDG cardiac uptake in mice subjected to short-term and long-term hypothyroidism, euthyroidism, and hyperthyroidism. Representative PET/CT images (**left**) and bar chart of mean cardiac voxel uptake normalized by mean whole-body voxel uptake (**right**). (**A**) Short-term (7 days) where (●): hypothyroid subjects, (▪); euthyroid subjects and (▲): hyperthyroid subjects, and (**B**) long-term (42 days) models. Data are presented as the mean ± SEM., ** *p* < 0.01.

At 7 and 42 days after the intervention, the mice exposed to hypothyroid (total thyroidectomy), physiological (sham), and hyperthyroid (T3 osmotic pump) conditions underwent [^18^F]-FDG PET/CT imaging to examine whether the transcriptional alterations observed in vitro in CSCs translate into functional metabolic consequences in vivo and if the response patterns are similar (Figure 4).

At the 7-day time point, cardiac [^18^F]-FDG uptake was significantly higher in the hypothyroid group compared to the control group (*p* < 0.01), indicating an acute increase in cardiac glucose uptake.

On the other hand, in hyperthyroid animals, glucose uptake decreased in comparison to the control group, mostly due to the shift toward oxidative phosphorylation rather than glycolysis. At 42 days, the hypothyroid and hyperthyroid groups’ cardiac FDG uptake converged toward the physiological levels observed in controls, indicating that metabolic adaptation occurs during chronic T3 dysregulation.

This temporal pattern aligns with that in the transcriptomic findings from the in vitro investigation, which showed that hypo- and hyperthyroidism significantly altered CSC gene expression after 24 h, but many of those changes disappeared or were reversed by 120 h.

## 4. Discussion

This study shows that hypothyroidism and hyperthyroidism affect the proliferation and expression of extracellular matrix (ECM)-related and growth factor genes in CSCs. These changes were mostly biphasic, i.e., the acute effects were attenuated or reversed after long-term exposure. PET imaging of hypothyroid and hyperthyroid mice showed that abnormal levels of thyroid hormone cause an acute change in cardiac glucose uptake that is followed by adaptation if they are chronically exposed to abnormal T3 levels. The concordance between the CSC and cardiac metabolic response patterns to abnormal thyroid levels suggest that CSCs are involved in the modulation of the cardiac metabolic response to altered thyroid levels.

Our data adds to the current knowledge on T3’s effects on cardiomyocytes and the heart as a whole [20] (Yamakawa et al., 2021) as the role of CSCs is understudied.

While standard quantitative assays (e.g., MTT, CellTiter-Glo, PicoGreen, etc.) can more accurately assess cell proliferation, we used cell counting as a preliminary exploratory approach as a proof of concept, and our future research will include standard proliferation assays. Previous studies on cardiac fibroblasts used the MTT assay and cell counting to evaluate their proliferation, and these two approaches produced similar trends. In the study of Zhang et al., after a short period of exposure to T3 dysregulation, pro-fibrotic genes like COL1A1 and COL3A1 were significantly upregulated. After 120 h, these changes mostly disappeared, indicating adaptation. Research by de Rycker et al., [14] (de Rycker et al., 1984) and Yao and Eghbali [24] (Yao and Eghbali 1992) has shown that normal levels of T3 can reduce the production of collagen by dermal fibroblasts and cardiac fibroblasts, respectively. These results support the idea that T3 has an antifibrotic effect and they do not contradict our findings. In our study, the euthyroid group had the lowest levels of collagen gene expression compared to the hypo- and hyperthyroid groups. This biphasic pattern shows that acute changes in either direction (high or low T3 levels) can be pro-fibrotic, and normal levels of T3 help maintain ECM homeostasis. Ghose Roy et al., [25] (Ghose Roy et al., 2007) found that thyroid hormone treatment lowered TIMP-3 and TIMP-4 levels in the left ventricle, which is in agreement with our finding that TIMP 1–3 expression increases in the case of T3 deprivation.

Our results suggest that TGF-β and FGF2 may be the main players in the pro-fibrotic response in hyperthyroid states. Their mRNA expression was much higher in the hyperthyroid group early on, while PDGF-A expression was higher in the hypothyroid state. These different growth factor patterns suggest that the fibrotic outcome is the same in both thyroid states, but the signaling pathways that lead to it may be different.

Our discovery that supraphysiological T3 levels increase bFGF expression agrees with earlier work by Davis et al., [26] (Davis et al., 2004), which showed that T3 has proangiogenic effects through pathways that depend on fibroblast growth factors.

Interestingly, IGF1 gene expression substantially increased in the short term in both hypo- and hyperthyroid states, but it decreased to levels lower than that of the control after 120 h. This temporal inversion suggests that there was an initial compensatory response followed by a longer-term downregulation. A previous study found that hypothyroidism lowers IGF-1 mRNA levels in the heart, while hyperthyroidism may raise IGF-1 mRNA levels [27] (Thomas et al., 1993).

Our findings showing that short-term hypothyroidism leads to increased expression of multiple growth factors, including IGF1, HGF, and EGF, raise the interesting possibility that the temporary drop in local T3 levels after myocardial infarction [28] (von Hafe et al., 2019) may act as a defense mechanism by temporarily boosting the paracrine support function of cardiac stromal cells. However, our long-term data show that these growth factor effects fade over time.

Most of our RNA expression comparisons showed modest differences. However, even small changes in transcription may translate into physiological effects. In addition, the accumulation of small changes in different factors may aggregate to cause a large shift in biological behavior. Furthermore, the concordance between the in vitro gene expression patterns and in vivo cardiac metabolic responses supports the physiological significance of these observations.

We found that both hypo- and hyperthyroid conditions significantly increased CSC proliferation after 24 h. There are multiple possible explanations for this observation. First, T3 dysregulation (both hypo- and hyperthyroidism) may accelerate early cell cycle entry (G1-to-S phase transition) by activating cyclin D1. Pibiri et al., [29] (Pibiri et al., 2001) demonstrated that T3 activates cyclin D1, an early target in hepatocyte proliferation.

Second, hypothyroidism causes paradoxical proliferation. Hirose et al., [30] (2019) demonstrated that inhibition of thyroid hormone signaling increases cardiac cell proliferation and regenerative capacity, primarily due to reduced oxidative phosphorylation which leads to lower levels of reactive oxygen species and less cell cycle arrest [30] (Hirose et al., 2019). Mogharbel et al., [31] (Mogharbel et al., 2017) found that hypothyroid mice had more bone marrow mesenchymal stem cells.

Our in vivo PET/CT data shows that acute T3-driven cardiac metabolic responses return to normal after a long period of exposure. Short-term hyperthyroidism decreased the uptake of FDG, which is in line with a metabolic shift away from glycolysis and toward fatty acid oxidation. On the other hand, hypothyroidism was linked to more glycolytic dependence and less oxidative capacity, as shown by the lower glucose uptake. The fact that these effects disappeared after 42 days supports the idea that adaptation mechanisms help restore cardiac homeostasis over time. These results are in line with those of our transcriptomic studies. Studies like [32] (Degens et al., 2003) have shown that T3 supplementation first improves heart function, as shown by an increased ejection fraction, but that this effect fades over time. Yao and Eghbali [24] (Yao and Eghbali 1992) also showed that T3-induced fibrotic signaling was only temporary by noting that collagen mRNA levels in the ventricular tissue peaked 24 h after T3 treatment and then went back to normal. These results support our metabolic and transcriptomic results.

The reversal/adaptation pattern we observed in vivo and in vitro can be explained by several mechanisms. First, thyroid hormone receptor (TR) signaling can change over time. For example, fibroblasts that are exposed to hormones for a long period of time show reduced expression and sensitivity of thyroid hormone receptors, which could lower transcriptional output even though the hormonal levels do not change [33] (Gunin et al., 2019). Second, the local regulation of TH by deiodinase enzymes (DIO1, DIO2, and DIO3) offers a robust mechanism for the temporal modulation of signaling by regulate intracellular T3 levels [34,35] (Bianco and Kim 2006; Dentice et al., 2013). Finally, fibroblast phenotypic switching, such as transitioning to myofibroblasts, can significantly reconfigure hormone responsiveness, including modified expression of TH-interacting integrins and deiodinases, resulting in context-dependent variations in TH sensitivity [36] (Kohon et al., 2023). These mechanisms collectively explain the diminishing influence of TH signaling in fibroblasts following extended exposure.

The finding that cardiac fibroblast and metabolic responses to altered thyroid hormone status are transient and adaptation occurs over time has important clinical implications. Several clinical studies suggest that TH supplementation after ischemic injury or heart failure can improve cardiac function [3,32] (Degens et al., 2003; Shi et al., 2022); our findings indicate that the duration of this treatment needs to be optimized to obtain the best outcomes. Conversely, experimental work by Hirose et al., [30] (Hirose et al., 2019) demonstrated that thyroid hormone deprivation enhances cardiac regeneration, highlighting that transient hypothyroid conditions may be beneficial, as shown by the increased growth factor expression observed in our study, but the duration also should be considered. Both hyper- and hypothyroidism affect pro-fibrotic gene expression, albeit through distinct mechanisms, emphasizing the need for precise dosing to avoid maladaptive fibrotic outcomes.

Even though we cannot definitively connect the in vivo metabolic normalization to CSC-specific mechanisms, this idea is supported by the fact that the in vitro CSC responses and in vivo imaging results show the same temporal patterns. Mechanical insights from CSCs point to the possibility that CSCs are involved in the metabolic adaptation of the heart to T3 dysregulation.

## 5. Conclusions

Our results clearly show that the timing and dose of T3 exposure must be considered when examining the effects of T3 on cardiac remodeling, proliferation, and paracrine signaling. This goes against the idea that thyroid hormones only have binary effects. The differential response of CSCs to different T3 concentrations and different T3 exposure durations could explain why earlier studies found contradictory results.

## Figures and Tables

**Table 1:** List of primers.

Gene	Forward (5′-3′)	Reverse (5′-3′)
ACTB	CATGTACGTTGCTATCCAGGC	CTCCTTAATGTCACGCACGAT
COL1A1	GAGGGCCAAGACGAAGACATC	CAGATCACGTCATCGCACAAC
COL3A1	TTGAAGGAGGATGTTCCCATCT	ACAGACACATATTTGGCATGGTT
COL4A1	GGACTACCTGGAACAAAAGGG	GCCAAGTATCTCACCTGGATCA
FN1	CGGTGGCTGTCAGTCAAAG	AAACCTCGGCTTCCTCCATAA
MMP2	CCCACTGCGGTTTTCTCGAAT	CAAAGGGGTATCCATCGCCAT
TIMP1	CTTCTGCAATTCCGACCTCGT	ACGCTGGTATAAGGTGGTCTG
TIMP2	AAGCGGTCAGTGAGAAGGAAG	GGGGCCGTGTAGATAAACTCTAT
TIMP3	CATGTGCAGTACATCCATACGG	CATCATAGACGCGACCTGTCA
TGFB1	TACCTGAACCCGTGTTGCTCTC	GTTGCTGAGGTATCGCCAGGAA
PDGFA	GCAAGACCAGGACGGTCATTT	GGCACTTGACACTGCTCGT
FGF2	AGAAGAGCGACCCTCACATCA	CGGTTAGCACACACTCCTTTG
IGF1	GCTCTTCAGTTCGTGTGTGGA	GCCTCCTTAGATCACAGCTCC
HGF	GCTATCGGGGTAAAGACCTACA	CGTAGCGTACCTCTGGATTGC
VEGFA	AGGGCAGAATCATCACGAAGT	AGGGTCTCGATTGGATGGCA
EGF	AGGCACGAGTAACAAGCTCAC	ATGAGGACATAACCAGCCACC

## Data Availability

The original contributions presented in this study are included in the article. Further inquiries can be directed to the corresponding authors.

## References

[1] Razvi S., Jabbar A., Pingitore A., Danzi S., Biondi B., Klein I., Peeters R., Zaman A., Iervasi G. (2018). Thyroid Hormones and Cardiovascular Function and Diseases. J. Am. Coll. Cardiol..

[2] Sato Y., Yoshihisa A., Kimishima Y., Kiko T., Kanno Y., Yokokawa T., Abe S., Misaka T., Sato T., Oikawa M. (2019). Low T3 Syndrome Is Associated with High Mortality in Hospitalized Patients with Heart Failure. J. Card. Fail..

[3] Shi C., Bao Y., Chen X., Tian L. (2022). The Effectiveness of Thyroid Hormone Replacement Therapy on Heart Failure and Low-Triiodothyronine Syndrome: An Updated Systematic Review and Meta-Analysis of Randomized Controlled Trials. Endocr. Pract..

[4] Osuna P.M., Udovcic M., Sharma M.D. (2017). Hypothyroidism and the Heart. Methodist DeBakey Cardiovasc. J..

[5] Al-Ghamdi A.S., Aljohani N. (2013). Graves’ Thyrotoxicosis-Induced Reversible Cardiomyopathy: A Case Report. Clin. Med. Insights Case Rep..

[6] Choudhury R.P., MacDermot J. (1998). Heart Failure in Thyrotoxicosis, an Approach to Management. Br. J. Clin. Pharmacol..

[7] Song X., Nie L., Long J., Zhao J., Liu X., Wang L., Liu D., Wang S., Liu S., Yang J. (2023). Hydrogen Sulfide Alleviates Hypothyroidism-Induced Myocardial Fibrosis in Rats through Stimulating Autophagy and Inhibiting TGF-Β1/Smad2 Pathway. Korean J. Physiol. Pharmacol..

[8] Liu M., Li Z., Liang B., Li L., Liu S., Tan W., Long J., Tang F., Chu C., Yang J. (2018). Hydrogen Sulfide Ameliorates Rat Myocardial Fibrosis Induced by Thyroxine through PI3K/AKT Signaling Pathway. Endocr. J..

[9] Bano A., Chaker L., Muka T., Mattace-Raso F.U.S., Bally L., Franco O.H., Peeters R.P., Razvi S. (2020). Thyroid Function and the Risk of Fibrosis of the Liver, Heart, and Lung in Humans: A Systematic Review and Meta-Analysis. Thyroid.

[10] Nicolini G., Pitto L., Kusmic C., Balzan S., Sabatino L., Iervasi G., Forini F. (2013). New Insights into Mechanisms of Cardioprotection Mediated by Thyroid Hormones. J. Thyroid. Res..

[11] Ojamaa K. (2010). Signaling Mechanisms in Thyroid Hormone-Induced Cardiac Hypertrophy. Vasc. Pharmacol..

[12] Araujo A., Diniz G., Seibel F., Branchini G., Ribeiro M., Brum I., Khaper N., Barreto-Chaves M., Belló-Klein A. (2011). Reactive Oxygen and Nitrogen Species Balance in the Determination of Thyroid Hormones-Induced Cardiac Hypertrophy Mediated by Renin–Angiotensin System. Mol. Cell. Endocrinol..

[13] Weltman N.Y., Ojamaa K., Schlenker E.H., Chen Y.-F., Zucchi R., Saba A., Colligiani D., Rajagopalan V., Pol C.J., Gerdes A.M. (2014). Low-Dose T3 Replacement Restores Depressed Cardiac T3 Levels, Preserves Coronary Microvasculature and Attenuates Cardiac Dysfunction in Experimental Diabetes Mellitus. Mol. Med..

[14] de Rycker C., Vandalem J.-L., Hennen G. (1984). Effects of 3,5,3’-triiodothyronine on Collagen Synthesis by Cultured Human Skin Fibroblasts. FEBS Lett..

[15] Murata Y., Ceccarelli P., Refetoff S., Horwitz A.L., Matsui N. (1987). Thyroid Hormone Inhibits Fibronectin Synthesis by Cultured Human Skin Fibroblsts. J. Clin. Endocrinol. Metab..

[16] Smith T.J., Murata Y., Horwitz A.L., Philipson L., Refetoff S. (1982). Regulation of Glycosaminoglycan Synthesis by Thyroid Hormone in Vitro. J. Clin. Investig..

[17] Schmohl K.A., Mueller A.M., Dohmann M., Spellerberg R., Urnauer S., Schwenk N., Ziegler S.I., Bartenstein P., Nelson P.J., Spitzweg C. (2019). Integrin Avβ3-Mediated Effects of Thyroid Hormones on Mesenchymal Stem Cells in Tumor Angiogenesis. Thyroid.

[18] Safer J.D., Crawford T.M., Fraser L.M., Hoa M., Ray S., Chen T.C., Persons K., Holick M.F. (2003). Thyroid Hormone Action on Skin: Diverging Effects of Topical versus Intraperitoneal Administration. Thyroid.

[19] Tallquist M.D., Molkentin J.D. (2017). Redefining the Identity of Cardiac Fibroblasts. Nat. Rev. Cardiol..

[20] Yamakawa H., Kato T.S., Noh J.Y., Yuasa S., Kawamura A., Fukuda K., Aizawa Y. (2021). Thyroid Hormone Plays an Important Role in Cardiac Function: From Bench to Bedside. Front. Physiol..

[21] Li T.-S., Cheng K., Lee S.-T., Matsushita S., Davis D., Malliaras K., Zhang Y., Matsushita N., Smith R.R., Marbán E. (2010). Cardiospheres Recapitulate a Niche-Like Microenvironment Rich in Stemness and Cell-Matrix Interactions, Rationalizing Their Enhanced Functional Potency for Myocardial Repair. Stem Cells.

[22] Larsen P.R., Kronenberg H.M., Melmed S., Polonsky K.S. (2003). Williams Textbook of Endocrinology.

[23] Moeller L.C., Dumitrescu A.M., Walker R.L., Meltzer P.S., Refetoff S. (2005). Thyroid Hormone Responsive Genes in Cultured Human Fibroblasts. J. Clin. Endocrinol. Metab..

[24] Yao J., Eghbali M. (1992). Decreased Collagen Gene Expression and Absence of Fibrosis in Thyroid Hormone-Induced Myocardial Hypertrophy. Response of Cardiac Fibroblasts to Thyroid Hormone in Vitro. Circ. Res..

[25] Roy S.G., Mishra S., Ghosh G., Bandyopadhyay A. (2007). Thyroid Hormone Induces Myocardial Matrix Degradation by Activating Matrix Metalloproteinase-1. Matrix Biol..

[26] Davis F.B., Mousa S.A., O’cOnnor L., Mohamed S., Lin H.-Y., Cao H.J., Davis P.J. (2004). Proangiogenic Action of Thyroid Hormone Is Fibroblast Growth Factor–Dependent and Is Initiated at the Cell Surface. Circ. Res..

[27] Thomas M.R., Miell J.P., Taylor A.M., Ross R.J.M., Arnao J.R., Jewitt D.E., McGregor A.M. (1993). Endocrine and Cardiac Paracrine Actions of Insulin-like Growth Factor-I (IGF-I) during Thyroid Dysfunction in the Rat: Is IGF-I Implicated in the Mechanism of Heart Weight/Body Weight Change during Abnormal Thyroid Function?. J. Mol. Endocrinol..

[28] von Hafe M., Neves J.S., Vale C., Borges-Canha M., Leite-Moreira A. (2019). The Impact of Thyroid Hormone Dysfunction on Ischemic Heart Disease. Endocr. Connect..

[29] Pibiri M., Ledda-Columbano G.M., Cossu C., Simbula G., Menegazzi M., Shinozuka H., Columbano A. (2001). Cyclin D1 Is an Early Target in Hepatocyte Proliferation Induced by Thyroid Hormone (T3). FASEB J..

[30] Hirose K., Payumo A.Y., Cutie S., Hoang A., Zhang H., Guyot R., Lunn D., Bigley R.B., Yu H., Wang J. (2019). Evidence for Hormonal Control of Heart Regenerative Capacity during Endothermy Acquisition. Science.

[31] Mogharbel B.F., Abdelwahid E., Irioda A.C., Francisco J.C., Simeoni R.B., De Souza D., De Souza C.M.C.O., Beltrame M.P., Ferreira R.J., Guarita-Souza L.C. (2017). Bone Marrow-Derived Stem Cell Populations Are Differentially Regulated by Thyroid or/and Ovarian Hormone Loss. Int. J. Mol. Sci..

[32] Degens H., Gilde A.J., Lindhout M., Willemsen P.H.M., van der Vusse G.J., van Bilsen M. (2003). Functional and Metabolic Adaptation of the Heart to Prolonged Thyroid Hormone Treatment. Am. J. Physiol. Heart Circ. Physiol..

[33] Gunin A.G., Golubtsova N.N., Kravtsova O.A., Subbotkin A.S., Subbotkina N.O., Filippov F.N. (2019). Number, Proliferative Activity, and Expression of Thyroid Hormone Receptors in Dermal Fibroblasts in Mice with Changed Thyroid Status. Bull. Exp. Biol. Med..

[34] Bianco A.C., Kim B.W. (2006). Deiodinases: Implications of the Local Control of Thyroid Hormone Action. J. Clin. Investig..

[35] Dentice M., Marsili A., Zavacki A., Larsen P.R., Salvatore D. (2013). The Deiodinases and the Control of Intracellular Thyroid Hormone Signaling during Cellular Differentiation. Biochim. Biophys. Acta (BBA)—Gen. Subj..

[36] Kohon M.Y., Levy M.Z., Hornik-Lurie T., Shalom A., Berl A., Drucker L., Levy Y., Matalon S.T. (2023). Avβ3 Integrin as a Link between the Development of Fibrosis and Thyroid Hormones in Systemic Sclerosis. Int. J. Mol. Sci..

